# Risk factors for photic phenomena in two different multifocal diffractive intraocular lenses

**DOI:** 10.1038/s41598-024-83838-x

**Published:** 2025-01-02

**Authors:** Yuki Ukai, Tsuyoshi Mito, Yui Nakatsugawa, Yusuke Seki, Norihiro Mita, Eri Shibuya, Mai Yamazaki, Eri Kubo, Hiroshi Sasaki

**Affiliations:** Department of Ophthalmology, Kanazawa Medical University, Kahoku, Ishikawa Japan

**Keywords:** Intraocular lens, Photic phenomena, Cataract surgery, Eye shape, Pupil size, Risk factors, Optics and photonics

## Abstract

Photic phenomena are more pronounced in presbyopia-corrected than in monofocal intraocular lens (IOL), causing dissatisfaction after cataract surgery. Photic Phenomena Test (PPT) quantifies photic phenomena in eyes with two types of presbyopia-corrected IOL. We examined the relationship between preoperative eye shape and pupil diameter. We included patients with PanOptix IOLs (PanOptix group, n = 38; 65.7 ± 9.2 years old) and Synergy IOLs (Synergy group, n = 39; 61.9 ± 9.6 years old), who underwent the PPT between 1 and 3 months after cataract surgery, from January 2021 to April 2023. The relationships between age, sex, pupil diameter, and higher-order corneal aberrations were examined and mean values for PPT measurements were compared between the groups. There was no difference in glare between the two groups. The halo was larger and thicker, and the starburst was larger and stronger in the Synergy group (P < 0.01). Postoperative halo brightness was positively correlated with the corneal coma aberration in the PanOptix group (P < 0.05). The Synergy group showed a positive correlation between the size and brightness of the postoperative halo and starburst and pupil diameter (P < 0.01). PPT, thus, revealed risk factors in eyes with two types of presbyopia-corrected IOL, which can be examined before cataract surgery to provide critical information for IOL selection.

## Introduction

Photic phenomena are more frequently perceived in eyes implanted with presbyopia-correcting intraocular lenses (IOLs) than in those implanted with monofocal IOLs^1^. Some adverse events, such as IOL exchange, have been reported, resulting in postoperative patient dissatisfaction^2–5^. The aggravation of photic phenomena in phakic eyes has been associated with pupil dilation and increased spherical aberration^6^. Preoperative identification of risk factors and a reduction in postoperative photic phenomena enable better patient satisfaction with presbyopia-correcting IOLs. We previously reported a quantitative and objective assessment method for photic phenomena using the photic phenomena test (PPT)^7^. We elucidated that the shape and degree of patients’ perception of photic phenomena vary according to the IOL type.

Quantifying the patients’ perception of glares, halos, and starbursts enables the analysis of their correlation with ocular shape and higher-order aberrations. In the present study, we evaluated the associations among preoperative ocular shape, pupil size, and postoperative photic phenomena to predict photic phenomena preoperatively in eyes implanted with two types of presbyopia-correcting IOLs.

## Results

The PanOptix group included 38 cases (38 eyes, mean age 65.7 ± 9.2 years) and the Synergy group included 39 cases (39 eyes, mean age 61.9 ± 9.6 years). Table 1 shows the demographic and preoperative clinical data of the two groups. Pupil size, various higher-order aberrations, manifest refractive spherical equivalent (MRSE), corrected visual acuity, and IOL power were comparable between the groups. However, the Synergy group had a significantly longer axial length. Table 2 shows the postoperative pupil size, various higher-order aberrations, MRSE, and corrected visual acuity at PPT measurements for both groups. No statistically significant differences were observed for any of the parameters, suggesting that the two groups were similar.

**Table 1:** Demographics and preoperative clinical data.

Characteristics	PanOptix group	Synergy group	P-value
Number of eyes	38	39	–
Age (Mean ± SD)	65.7 ± 9.2	61.9 ± 9.6	0.086
Age (Range)	36–79	44–77	–
Male/Female (n)	14/24	17/22	0.644
Photopic pupil size (mm)	3.10 ± 0.43	3.29 ± 0.50	0.094
Total higher order aberration (μm)	0.15 ± 0.05	0.15 ± 0.07	0.988
Coma aberration (μm)	0.09 ± 0.04	0.09 ± 0.05	0.977
Spherical aberration (μm)	0.05 ± 0.03	0.06 ± 0.03	0.501
MRSE (D)	-3.03 ± 3.93	-4.85 ± 4.77	0.073
Corrected visual acuity (logMAR)	0.14 ± 0.16	0.27 ± 0.40	0.077
Axial length (mm)	24.54 ± 1.44	25.58 ± 1.98	0.011
IOL power (D)	17.03 ± 4.46	14.85 ± 5.67	0.065

Total higher-order aberrations, coma aberrations, and spherical aberrations were measured at a diameter of 4 mm for corneal higher-order aberrations. IOL, MRSE, SD, MAR.

**Table 2:** Postoperative clinical data.

	PanOptix group	Synergy group	P-value
Pupil size measured using the PPT (mm)	3.95 ± 0.77	4.07 ± 0.64	0.448
Total higher-order aberration (μm)	0.15 ± 0.06	0.15 ± 0.07	0.947
Coma aberration (μm)	0.08 ± 0.04	0.08 ± 0.06	0.872
Spherical aberration (μm)	0.05 ± 0.02	0.06 ± 0.03	0.350
MRSE (D)	0.04 ± 0.33	0.15 ± 0.27	0.090
Corrected visual acuity (logMAR)	-0.11 ± 0.07	-0.13 ± 0.07	0.359

Total higher-order aberrations, coma aberrations, and spherical aberrations were measured at a diameter of 4 mm for corneal higher-order aberrations. MRSE, MAR, PPT.

**Fig. 1:**
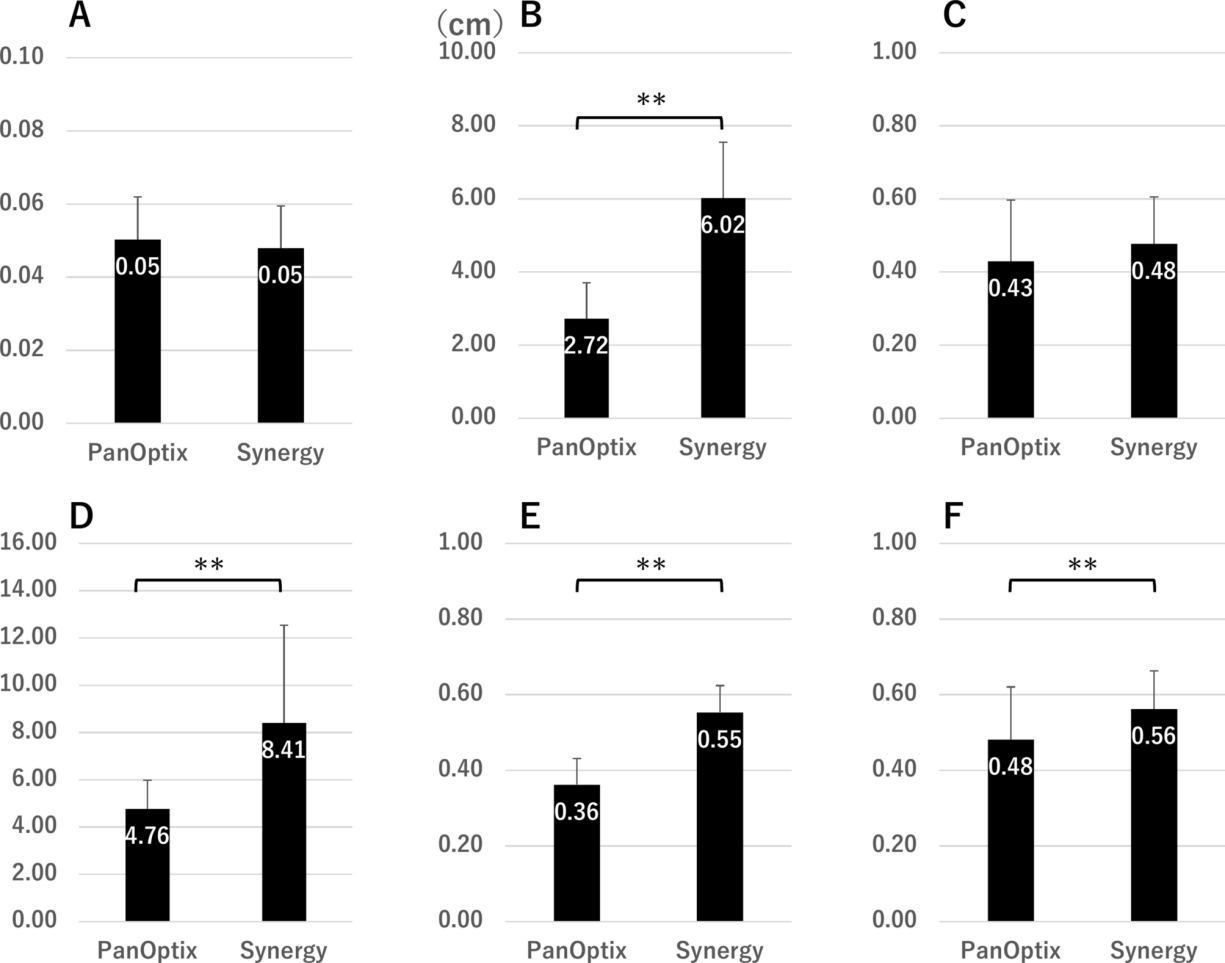
Mean value of the (**A**) glare size, (**B**) halo size, (**C**) halo intensity, (**D**) halo ring width, (**E**) starburst size, and (**F**) starburst intensity for each IOL group. **P < 0.01 IOL, intraocular lens.

Figure 1 shows the size of the glare, halo, and starbursts; the intensity of the halo and starburst; and the ring width of the halo measured using PPT for the PanOptix and Synergy groups. In the Synergy group, the halos were larger and thicker. The starbursts were also larger and more intense.

Tables 3 and 4 present the results of the simple regression analysis of various parameters and PPT outcomes in both groups. In the PanOptix group, a significant positive correlation was observed between halo brightness and coma aberration, indicating a stronger perception of halo with increased coma aberrations. No significant correlations were found for the size of the glare, halo, starbursts, or halo thickness. In the Synergy group, significant positive correlations were observed between the halo size, halo brightness, starburst size, starburst brightness, and pupil size. This suggests that stronger and larger halos and starbursts were perceived to be associated with larger pupil sizes. Furthermore, the halo size showed a significant negative correlation with age, indicating that older adults perceived smaller halos than younger participants.

**Table 3:** Univariate analysis of the PanOptix group.

Objective variable	Explanatory variable	Regression coefficients	95% CI lower bound	95% CI upper bound	Standard error	P-value
Glare size	Sex (male = 0, Female = 1)	0.00381	− 0.00420	0.01182	0.00395	0.341
	Age	0.00024	− 0.00018	0.00066	0.00021	0.259
	Pupil size (mm)	− 0.00345	− 0.00845	0.00155	0.00247	0.171
	Higher-order Aberrations (μm)	0.02802	− 0.03755	0.09360	0.03233	0.392
	Coma aberrations (μm)	0.00471	− 0.09349	0.10292	0.04842	0.923
	Spherical aberrations (μm)	− 0.03690	− 0.18952	0.11572	0.07525	0.627
Halo size	Sex (male = 0, Female = 1)	− 0.15893	− 0.83469	0.51683	0.33320	0.636
	AGE	− 0.01391	− 0.04946	0.02165	0.01753	0.433
	Pupil size (mm)	0.26369	− 0.15583	0.68320	0.20685	0.211
	Higher- order Aberrations (μm)	1.45783	− 4.05440	6.97006	2.71794	0.595
	Coma aberrations (μm)	− 1.78622	− 9.96753	6.39509	4.03399	0.661
	Spherical aberrations (μm)	− 11.75129	− 23.90922	0.40665	5.99476	0.058
Halo intensity	Sex (male = 0, Female = 1)	− 0.01185	− 0.12792	0.10423	0.05723	0.837
	Age	− 0.00003	− 0.00618	0.00611	0.00303	0.991
	Pupil size (mm)	0.02095	− 0.05219	0.09408	0.03606	0.565
	Higher− order Aberrations (μm)	0.30427	− 0.63833	1.24687	0.46477	0.517
	Coma aberrations (μm)	1.42571	0.10536	2.74605	0.65103	0.035
	Spherical aberrations (μm)	1.50919	− 0.62201	3.64040	1.05084	0.160
Halo ring width	Sex (male = 0, Female = 1)	− 0.60119	− 1.42289	0.22051	0.40516	0.147
	Age	− 0.00538	− 0.05012	0.03937	0.02206	0.809
	Pupil size (mm)	0.18094	− 0.35108	0.71296	0.26232	0.495
	Higher- order Aberrations (μm)	− 0.29373	− 7.20337	6.61591	3.40696	0.932
	Coma aberrations (μm)	0.95142	− 9.28699	11.18984	5.04829	0.852
	Spherical aberrations (μm)	2.45870	− 13.49047	18.40787	7.86412	0.756
Starburst size	Sex (male = 0, Female = 1)	− 0.00387	− 0.05167	0.04393	0.02357	0.871
	Age	− 0.00123	− 0.00372	0.00127	0.00123	0.325
	Pupil size (mm)	0.01495	− 0.01488	0.04477	0.01471	0.316
	Higher-order Aberrations (μm)	0.14718	− 0.24003	0.53440	0.19093	0.446
	Coma aberrations (μm)	0.28125	− 0.28959	0.85209	0.28147	0.324
	Spherical aberrations (μm)	− 0.40016	− 1.29222	0.49191	0.43986	0.369
Starburst intensity	Sex (male = 0, Female = 1)	0.00435	− 0.09190	0.10059	0.04746	0.928
	Age	0.00285	− 0.00215	0.00785	0.00247	0.255
	Pupil size (mm)	0.00534	− 0.05553	0.06622	0.03001	0.860
	Higher− order Aberrations (μm)	− 0.15500	− 0.93907	0.62908	0.38661	0.691
	Coma aberrations (μm)	0.47898	− 0.67457	1.63253	0.56879	0.405
	Spherical aberrations (μm)	− 0.11870	− 1.93440	1.69701	0.89528	0.895

CI, confidence interval.

**Table 4:** Univariate analysis of the Synergy group.

Objective variable	Explanatory variable	Regression coefficients	95% CI lower bound	95% CI upper bound	Standard error	P− value
Glare size	Sex (male = 0, Female = 1)	0.00053	− 0.00710	0.00817	0.00377	0.888
	Age	0.00005	− 0.00035	0.00045	0.00020	0.814
	Pupil size (mm)	− 0.00232	− 0.00822	0.00359	0.00291	0.432
	Higher-ions (μm)	− 0.00312	− 0.05743	0.05119	0.02680	0.908
	Coma aberrations (μm)	− 0.00385	− 0.06959	0.06189	0.03245	0.906
	Spherical aberrations (μm)	0.06655	− 0.05048	0.18358	0.05776	0.257
Halo size	Sex (male = 0, Female = 1)	− 0.16176	− 1.17376	0.85023	0.49946	0.748
	Age	− 0.06231	− 0.11112	− 0.01350	0.02409	0.014
	Pupil size (mm)	1.04623	0.33711	1.75535	0.34998	0.005
	Higher-order Aberrations (μm)	3.81505	− 3.28410	10.91419	3.50369	0.283
	Coma aberrations (μm)	2.80966	− 5.86958	11.48890	4.28352	0.516
	Spherical aberrations (μm)	7.42933	− 8.18912	23.04778	7.70828	0.341
Halo intensity	Sex (male = 0, Female = 1)	0.06291	− 0.02001	0.14584	0.04093	0.133
	Age	− 0.00271	− 0.00709	0.00168	0.00216	0.219
	Pupil size (mm)	0.10496	0.04818	0.16175	0.02803	0.001
	Higher- order Aberrations (μm)	− 0.04805	− 0.65652	0.56042	0.30030	0.874
	Coma aberrations (μm)	− 0.46620	− 1.18641	0.25400	0.35545	0.198
	Spherical aberrations (μm)	− 0.06920	− 1.40361	1.26521	0.65858	0.917
Halo ring width	Sex (male = 0, Female = 1)	0.88503	− 1.83699	3.60705	1.34342	0.514
	Age	− 0.00865	− 0.15192	0.13462	0.07071	0.903
	Pupil size (mm)	− 0.93552	− 3.04732	1.17628	1.04225	0.375
	Higher- order Aberrations (μm)	− 8.81039	− 28.07239	10.45161	9.50650	0.360
	Coma aberrations (μm)	− 4.27020	− 27.81149	19.27109	11.61849	0.715
	Spherical aberrations (μm)	16.30542	− 26.06995	58.68079	20.91379	0.441
Starburst size	Sex (male = 0, Female = 1)	0.01008	− 0.03672	0.05688	0.02310	0.665
	Age	− 0.00191	− 0.00428	0.00046	0.00117	0.112
	Pupil size (mm)	0.04572	0.01245	0.07898	0.01642	0.008
	Higher- order Aberrations (μm)	− 0.06661	− 0.39977	0.26656	0.16443	0.688
	Coma aberrations (μm)	− 0.18059	− 0.58025	0.21907	0.19725	0.366
	Spherical aberrations (μm)	− 0.19238	− 0.92170	0.53694	0.35994	0.596
Starburst intensity	Sex (male = 0, Female = 1)	− 0.01618	− 0.08324	0.05089	0.03310	0.628
	Age	− 0.00154	− 0.00502	0.00194	0.00172	0.376
	Pupil size (mm)	0.06220	0.01401	0.11039	0.02378	0.013
	Higher−-order Aberrations (μm)	0.28380	− 0.18558	0.75318	0.23166	0.228
	Coma aberrations (μm)	0.22306	− 0.35171	0.79783	0.28367	0.437
	Spherical aberrations (μm)	0.06341	− 0.98622	1.11304	0.51803	0.903

CI, confidence interval.

**Fig. 2:**
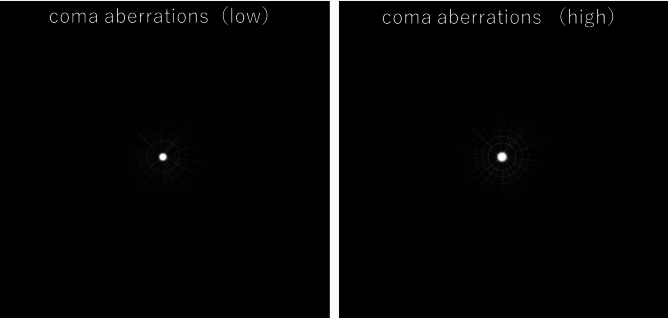
Typical photic phenomena of the PanOptix group.

**Fig. 3:**
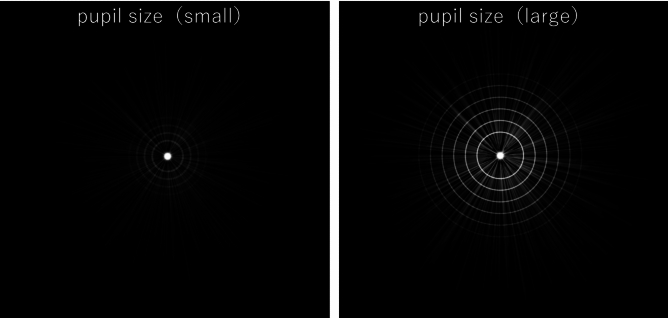
Typical photic phenomena of the Synergy group.

Tables 5 and 6 show the results of multiple regression analyses (stepwise backward method) of various parameters and PPT measurements in both groups. In the PanOptix group, corneal coma aberration was identified as a factor affecting postoperative halo brightness. In contrast, pupil size was identified as a factor affecting postoperative halo, starburst size, and brightness in the Synergy group. Figures 2 and 3 show representative images of photic phenomena using PPT in the PanOptix and Synergy groups, indicating that in the PanOptix group, patients with high corneal coma aberration perceived strong halos. Meanwhile, patients with larger pupil sizes in the Synergy group perceived larger and stronger halos and starbursts.

**Table 5:** Multiple regression analyses (stepwise backward method) in the PanOptix group.

Objective variable	Explanatory variable	Standardized partial regression coefficient	Correlation coefficient (r)	P-value
Glare size	None			
Halo size	None			
Halo intensity	Coma aberrations (μm)	0.43	0.34	0.02
Halo ring width	None			
Starburst size	None			
Starburst intensity	None

**Table 6:** Multiple regression analyses (stepwise backward method) in the Synergy group.

Objective variable	Explanatory variable	Standardized partial regression coefficient	Correlation coefficient (r)	P-value
Glare size	None			
Halo size	Pupil size	0.42	0.44	< 0.01
Halo intensity	Pupil size	0.48	0.52	< 0.01
Halo ring width	None			
Starburst size	Pupil size	0.34	0.42	< 0.01
Starburst intensity	Pupil size	0.55	0.39	< 0.01

**Fig. 4:**
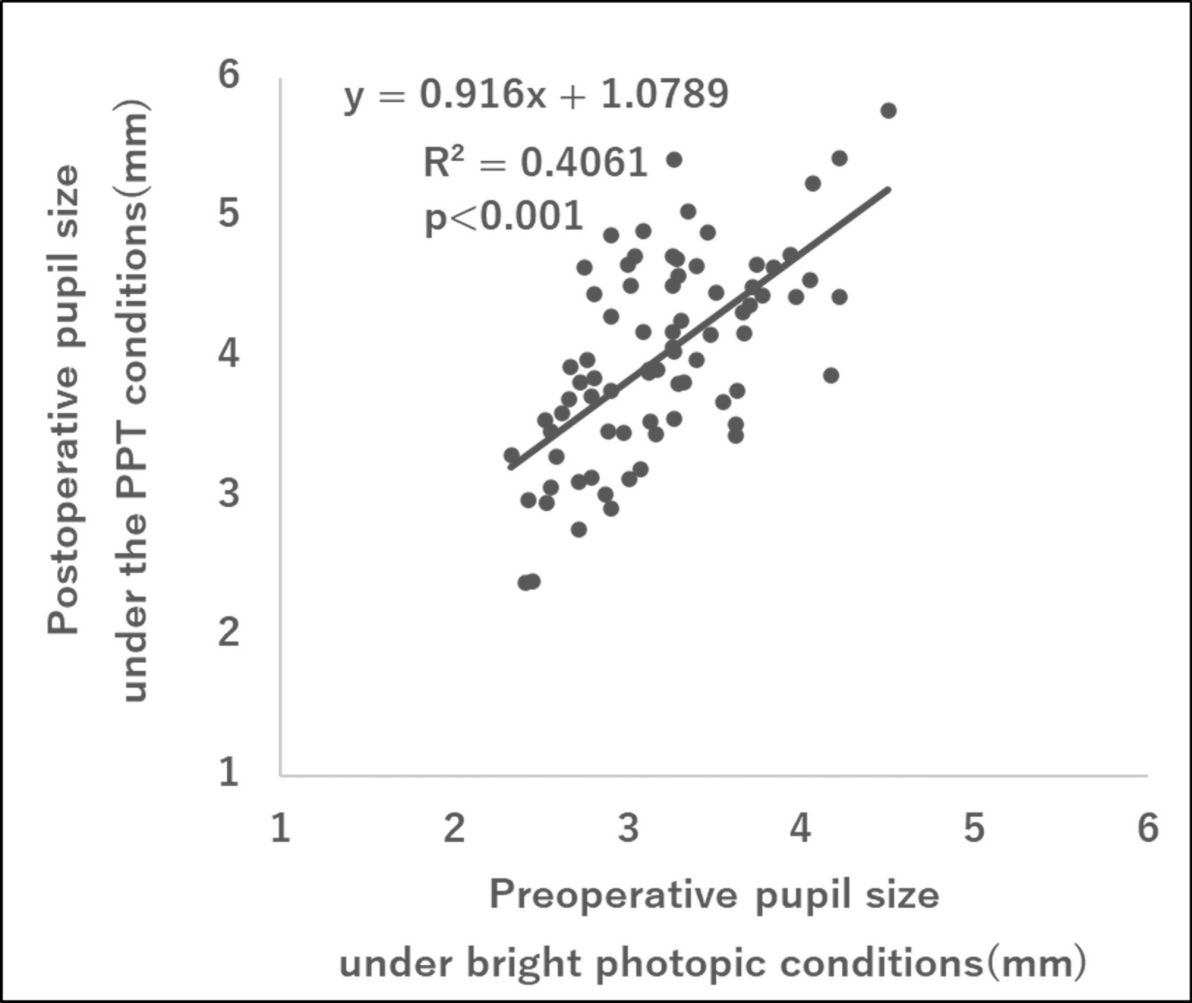
Correlation between preoperative pupil size under bright photopic conditions and postoperative pupil size under the PPT conditions in all cases of PanOptix and Synergy implantation. PPT, Photic Phenomena Test.

**Fig. 5:**
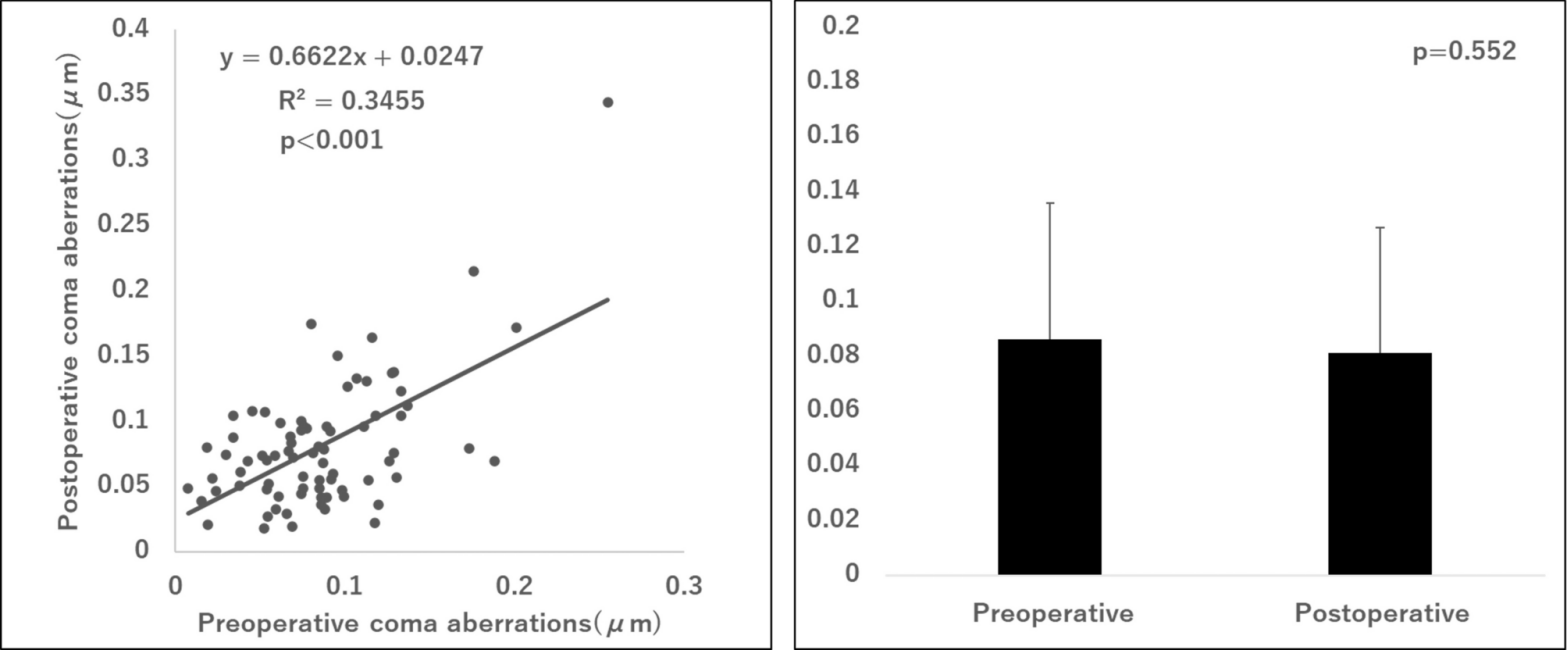
Correlation between preoperative and postoperative corneal coma aberrations in all cases of PanOptix and Synergy implantation.

Figure 4 shows the correlation between preoperative and postoperative pupil sizes under photopic and PPT conditions, respectively, for all cases of PanOptix and Synergy implantation. Figure 5 illustrates the correlation between preoperative and postoperative corneal coma aberrations in all cases of PanOptix and Synergy implantation. The postoperative pupil size under PPT conditions was significantly correlated with the preoperative photopic pupil size. Furthermore, there was a significant correlation between preoperative and postoperative corneal coma aberrations with a similar magnitude of aberration.

## Discussion

Our study identified the factors causing photic phenomena by analyzing the correlations between preoperative and postoperative ocular optical parameters with a quantitative assessment using PPT. In the PanOptix group, the risk factor for photic phenomena was corneal coma aberrations, while in the Synergy group, the risk factor was the pupil size.

**Fig. 6:**
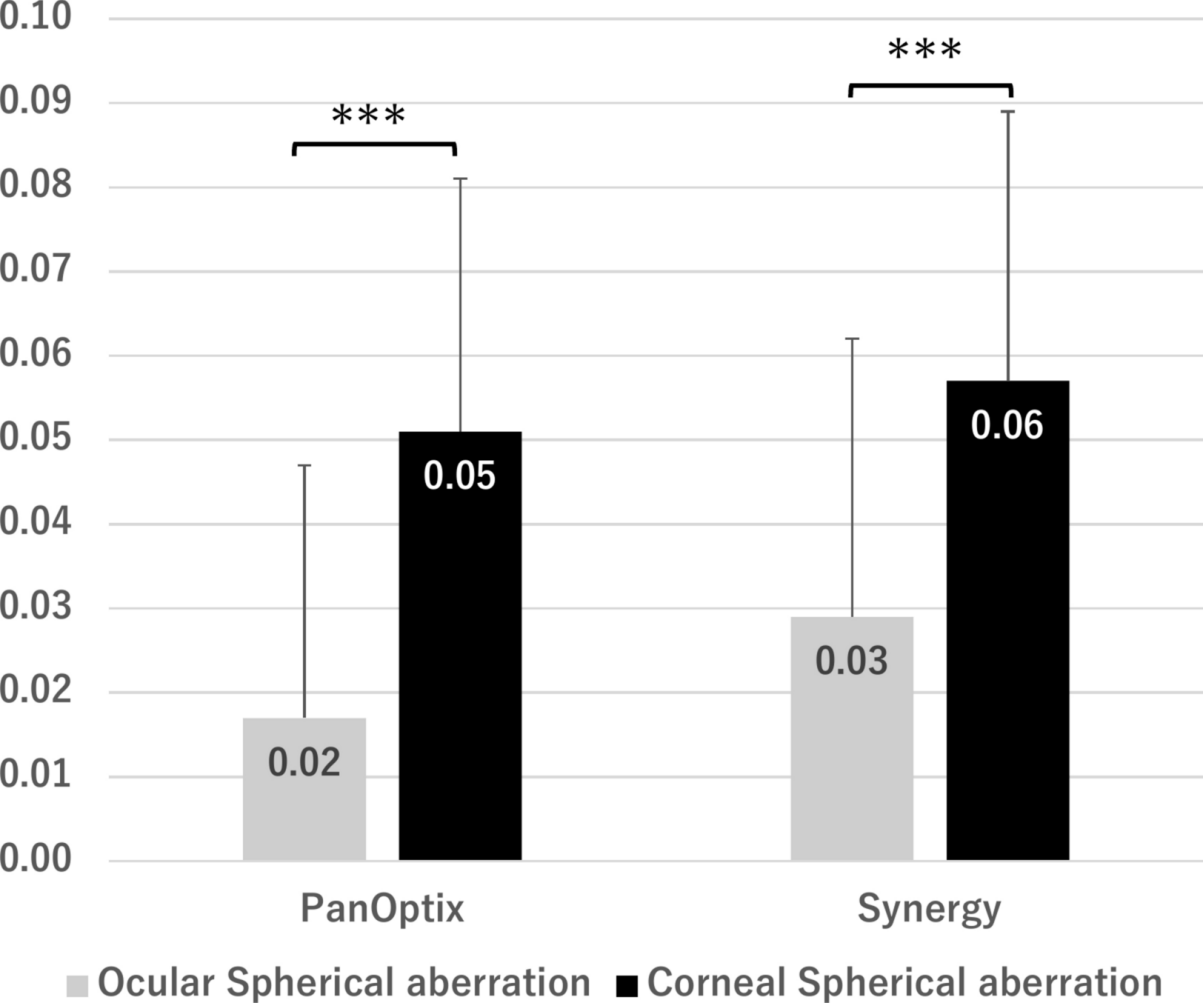
Corneal and ocular spherical aberrations after cataract surgery. ***P < 0.001.

We introduced the PPT as a quantitative measurement method for photic phenomena^7^. The PPT enables a highly reproducible assessment of photic phenomena in real time while presenting a light source in the examination room. Although previous studies have explored the factors related to photic phenomena^6,8,9^, many have evaluated the size of these photic phenomena using image analysis or a scale of approximately 10 levels. Unlike previous studies, our study employed a dedicated simulator that could vary the intensity of photic phenomena across 100 levels while directly exposing patients to a light source, creating an environment similar to daily life, and examining the correlation with pupil size under controlled conditions. Our findings revealed that among higher-order aberrations, only coma aberration was a risk factor for photic phenomena in the PanOptix group, with no significant correlation found for spherical aberrations. Macedo-de-Araújo et al. suggested that pupil size and spherical aberration in phakic eyes influence photic phenomena^6^. The target IOLs in our study had a non-spherical design. Figure 6 indicates that the postoperative ocular spherical aberration is significantly smaller than corneal spherical aberration. This may explain why spherical aberrations were not identified as a risk factor.

Additionally, some reports have suggested that coma aberration increases photophobia postoperatively^10^, potentially enhancing photic phenomena in eyes with PanOptix. As previously reported, there was no significant change in preoperative and postoperative corneal coma aberration^11^. Our results also revealed no significant differences between preoperative and postoperative coma aberrations. These results suggest the need for caution when using the PanOptix in patients with large preoperative corneal coma aberrations. Vega et al. reported a correlation between the halo and pupil size using image analysis with in vitro model eyes^9^. The study indicates that the AcrySof® IQ ACTIVEFOCUS (Alcon, Geneva, Switzerland), with it’s apodized structure, is minimally affected by pupil size. In contrast, the TECNIS ZKB00 (Johnson & Johnson Vision, Santa Ana, CA, USA), which features a diffractive region extending to the periphery of the IOL, tends to cause halos as the pupil size increases. In this study, the risk factors in the PanOptix and Synergy groups were different. In the PanOptix group, pupil expansion was not a significant risk factor, but halos and starbursts increased with pupil size in the Synergy group. Diffractive IOLs reportedly cause halos and starbursts^12,13^. PanOptix has a structure with an optical diameter of 6 mm and a diffractive region of 4.5 mm. Conversely, Synergy has an optical diameter of 6 mm and a diffractive region extending to the periphery of the IOL. PanOptix IOL can make the far-vision region dominant in cases with large pupils. However, in the Synergy group, the large diffractive region may have increased photic phenomena in patients with large pupils.

This study revealed a significant positive correlation between photic phenomena and pupil size under the PPT conditions in the Synergy group. Pupil size is not commonly evaluated in ophthalmic examinations under the PPT conditions. However, if a correlation between photopic pupil size and pupil size under PPT is observed, it would be possible to predict pupil size under PPT based on the photopic pupil size. A significant correlation was observed between the environmental pupil diameter at the time of postoperative PPT measurement and the photopic pupil diameter before surgery. In addition, Semsettin et al. reported a significant correlation between preoperative and postoperative changes in pupil size, preoperatively and postoperatively^14^. All risk factors detected in this study can be considered predictable parameters before surgery and can provide useful information for determining IOL indications in advance.

Our results revealed that the Synergy group experienced stronger photic phenomena compared to the PanOptix group. Similar results have been reported previously—patients with eyes implanted with Synergy IOLs have a higher risk of postoperative dissatisfaction^15^. The Synergy IOL is characterized by chromatic aberration correction and results in more pronounced glare and halos than the PanOptix IOL^15,16^. Our results indicate that the Synergy group exhibited significantly stronger halos, but there was no difference in glare perception. While there may be differences in the environment between actual nighttime driving conditions and the controlled setting of an examination room, it is possible that a more accurate evaluation of photic phenomena characteristics was achieved during testing with the PPT. Unlike during driving, where individuals cannot focus on light sources, the PPT enables participants to undergo the examination while actively fixating on the light source. This unique feature may enhance the precision of assessing photic phenomena. Indeed, an in vitro study also reported stronger photic phenomena associated with the Synergy IOL compared to the PanOptix IOL^17^, consistent with our current findings.

In eyes with the Synergy IOL, Moshirfar et al. reported dissatisfaction during nighttime driving at one month postoperatively^15^. Similarly, halos are a common visual disturbance, significantly impacting vision during nighttime driving^18^. Additionally, the Synergy IOL is associated with a high frequency of halos^19–21^. Baur et al. reported that in cases of Synergy implantation in patients undergoing refractive lens exchange (RLE), the majority experienced halos, but 71.4% did not find them bothersome^22^. In cases of RLE with few preoperative photic phenomena, patients with Synergy IOL experienced relatively low postoperative discomfort from halos. This suggests that while halos are commonly perceived with Synergy IOL, they are not severe enough to disrupt daily life.

Moreover, cases have been documented in which patients required IOL explantation, due to perceived halos, starbursts, and glare associated with the Synergy IOL^23^. Our findings similarly revealed pronounced photic phenomena with the Synergy IOL, particularly in cases with larger pupil diameters. These findings suggest that the selection of the Synergy IOL should be undertaken carefully.

As a countermeasure to the photic phenomenon, it may be necessary to remove or replace the IOL^24^. It will be extremely significant if it becomes possible to predict the photic phenomenon before surgery. In this study, we did not examine the predictive accuracy of postoperative PPT test results, so verifying this in the future is necessary. However, PanOptix IOL may cause strong postoperative photic phenomena in cases with large corneal coma aberration and Synergy IOL in patients with large pupil sizes. In addition, when the pupil diameter is smaller than a certain value, photic phenomenon may be mild even if Synergy is inserted. Synergy should be selected in patients with small pupils, even in those who need to drive at night. This study has limitations, including the use of pupil diameter at the time of PPT measurement post-surgery, and the inability to investigate the long-term risk of photic phenomena. Lubiński et al. reported that halos become lighter over time, suggesting a potential reduction in photic phenomena over the long term^25^. Long-term investigations are needed to see if the same trend is shown in the eyes at risk of photic phenomena. In this study, we confirmed the relationship between high-order aberrations and pupil diameter of the cornea and photic phenomena due to PPT by postoperative PPT examination. However, we did not investigate the relationship between photic phenomena in the patient’s daily life. In the future, we intend to investigate the relationship between preoperative coma aberration, pupil diameter, and postoperative photic phenomena in a prospective study that will include a PPT examination and a questionnaire survey of subjective symptoms, related to postoperative photic phenomena.

In conclusion, this study clarified the risk factors for postoperative photic phenomena in PanOptix- and Synergy IOL. Although it is necessary to verify the relationship between subjective symptoms related to photic phenomena and the results of the PPT examination, photic phenomena may be strongly felt in patients with large corneal coma aberration in eyes with PanOptix and in cases with large pupil size in eyes with Synergy.

## Methods

This prospective study included patients with PanOptix IOLs (AcrySof IQ PanOptix Trifocal; Alcon) or Synergy IOLs (TECNIS Synergy; Johnson & Johnson Vision), who consulted ophthalmologists at Kanazawa Medical University and underwent a quantitative assessment of photic phenomena using PPT, between 1 and 3 months after cataract surgery from January 2021 to April 2023. Patients with inflammatory conditions, such as corneal diseases, macular diseases, vitreous opacity, optic nerve diseases, uveitis, or a history of intraocular or extraocular surgery other than cataracts, were excluded. This study was conducted in compliance with the Declaration of Helsinki, and informed consent was obtained from all participants after they were given a thorough explanation. In addition, the study was approved by the Research Ethics Committee of Kanazawa Medical University (Approval Number: 1278).

All the surgeries were performed by experienced surgeons (HS, EK, and TS). Continuous curvilinear capsulorrhexis (CCC) and nuclear division were performed using a femtosecond laser (LenSx, Alcon). The main incision was a 1.5 mm long corneal incision created with a 2.2 mm blade (SharpCreation SL22; KAI INDUSTRIES Co. Ltd., Tokyo, Japan). After hydrodissection, the nuclear division was performed using a phaco chopper, followed by aspiration and fragmentation using CENTURION (Alcon) with an active entry (Alcon). A viscoelastic substance (DisCoVisc, Alcon) was used, and the IOL was inserted in the capsular bag. In all cases, the optical part of the IOLs was completely covered and fixed via the CCC. At the end of the surgery, the main incision and side ports were hydrated to ensure no leakage of the aqueous humor.

The PPT measurements were conducted according to a previous report^7^ using a white light-emitting diode with a luminance of 45,241 (cd/m^2^) as the light source. Additionally, pupil size was measured using VIP™-300 (NeurOptics, Irvine, CA, USA) to investigate the risk factors for photic phenomena.

Total higher-order aberrations, coma aberrations, and spherical aberrations at a 4 mm-analysis diameter were measured using KR-1W (TOPCON MEDICAL JAPAN Co. Ltd., Tokyo, Japan). Postoperative pupil size was measured in the twilight environment at a sunset illumination level of 7 lx while presenting the light source. The preoperative pupil size was measured using a WAM-5500 (Grand Seiko Co., Ltd., Tokyo, Japan) in a photopic environment, lit to a level of 500 lx.

Pearson’s correlation coefficient was used for univariate analysis to assess the correlation between the obtained PPT measured values (the size of glare, halo, and starbursts; the intensity of halo and starburst; and ring width of halo) and age, sex, pupil size, and corneal higher-order aberrations. A stepwise multiple regression analysis was conducted to identify significant risk factors. The comparison of each measurement and demographics between the two groups was conducted using a T-test or χ2 test. Furthermore, the sample size for the comparison of PPT measurements between the two groups was calculated based on each mean and standard deviation. We confirmed that the required sample size was met, with a statistical power of > 80%, which is within the acceptable range for statistical power^26^. All statistical analyses and sample size calculations were performed using the statistical software, Easy R version 1.37 (R Foundation for Statistical Computing, Vienna, Austria), and statistical significance was set at P < 0.05. Additionally, In multiple regression analysis, the Adjusted R-squared is calculated by statistical software, taking sample size into account, to assess model fit.

## Data Availability

All data supporting the findings of this study are available within the paper and its supplementary materials. The datasets generated and/or analyzed during the current study are available from the corresponding author on reasonable request. Hiroshi Sasaki Chief Professor of Ophthalmology in Kanazawa Medical University E-mail: mogu@kanazawa-med.ac.jp.
